# Finite Element Simulation for Analysing the Design and Testing of an Energy Absorption System

**DOI:** 10.3390/ma9080660

**Published:** 2016-08-05

**Authors:** Abraham Segade, José A. López-Campos, José R. Fernández, Enrique Casarejos, José A. Vilán

**Affiliations:** 1 Departamento de Ingeniería Mecánica, Universidade de Vigo, Campus As Lagoas Marcosende s/n, 36310 Vigo, Spain; asegade@uvigo.es (A.S.); joseangellopezcampos@uvigo.es (J.A.L.-C.); e.casarejos@uvigo.es (E.C.); jvilan@uvigo.es (J.A.V.); 2 Departamento de Matemática Aplicada I, Universidade de Vigo, ETSI Telecomunicación, Campus As Lagoas Marcosende s/n, 36310 Vigo, Spain

**Keywords:** finite element method, explicit dynamics, impact attenuator, energy absorption system, thermoplastic foam

## Abstract

It is not uncommon to use profiles to act as energy absorption parts in vehicle safety systems. This work analyses an impact attenuator based on a simple design and discusses the use of a thermoplastic material. We present the design of the impact attenuator and a mechanical test for the prototype. We develop a simulation model using the finite element method and explicit dynamics, and we evaluate the most appropriate mesh size and integration for describing the test results. Finally, we consider the performance of different materials, metallic ones (steel AISI 4310, Aluminium 5083-O) and a thermoplastic foam (IMPAXX500™). This reflects the car industry’s interest in using new materials to make high-performance, low-mass energy absorbers. We show the strength of the models when it comes to providing reliable results for large deformations and strong non-linearities, and how they are highly correlated with respect to the test results both in value and behaviour.

## 1. Introduction

There has been continual improvement in passive vehicle safety systems over recent decades. A better understanding of the plastic behaviour of materials has made it possible to design structures that are more efficient at absorbing the energy released in an impact. For conventional vehicles, about 50% of the energy absorption effects are located along the front rail of the car, which makes it a critical component and a challenge for safety engineers (see, for instance, [1,2]).

To better understand the energy absorption process, it is very important to study what is called “axial crushing” of the main metallic components. In the 1980s many studies analysed the behaviour of typical tubular structures under axial impact conditions [3,4,5]. Some of the models presented served as a basis to further develop vehicle energy absorption (EA) systems [6,7] based on the use of profiled structures. The most common element was the so-called “crash box” which connected the front panel of the car with the front rail structure, and was mostly made of steel.

Further studies into the behaviour of energy absorption (EA) systems introduced design improvements as well as mass reduction when this also became a goal for the industry. The use of high strength steel [8] helped to keep structural stiffness while reducing mass. Aluminium profiles made it possible to use simple geometries (circular, hexagonal) [9,10] as well as more complex multi-cells [11], which achieved an important mass reduction at the same time. The use of new materials can also help in the challenge to decrease total vehicle mass. These materials are either CFRPs [12], woven fiberglass/polyamide [13,14] or “cellular” (foam) materials [15]. Some proposals for ”hybrid” EA systems combine profiles and a filling of (metallic) foam or cells, using only aluminium: with simple tubular profiles [16,17], and with a double concentric tube separated either by a filling of foam [18,19] or honeycomb [19,20]. Other proposals combine materials: steel tube filled with CFRP (polymers), GFRP (polyamide), polyethylene terephthalate foam and cork conglomerates [21,22,23]. A further step in weight reduction can be achieved if the metallic parts are avoided by using only polymers and thermoplastics.

In this work, first we present an element designed as part of a car’s EA system. We show the measurements obtained as part of the tests performed on one prototype. In the third section we describe the finite element approximation we used to analyse the test results. In the final section, after the validation of the model, we study the performance of a thermoplastic foam as a possible material for achieving the right functionality in an EA system that can be applied in the automotive industry.

## 2. Design and Test of an Impact Attenuator

The structures used as elements of the EA system in race cars are extremely demanding. We studied an ‘impact attenuator’ following the specific rules of the race category which is known as Formula SAE. This is a student design competition which allows cars with engines up to 610 cc (maximum swept displacement) developed by university-based teams including both academic and under-graduated staff. The detailed rules [24] define the technical demands for structure, dynamics, engine, power and electrical systems and safety demands for the car. Our work was focused on the design of the system acting as impact attenuator (IA) in the car Driver’s Cell, which aims to provide driver safety of the driver in the event of a crash or impact. The rules define the minimum dimensions of the attenuator, how it is fixed to the car structure, and the test conditions for it to be evaluated as a single piece.

### 2.1. Impact Attenuator Design

**Figure 1:**
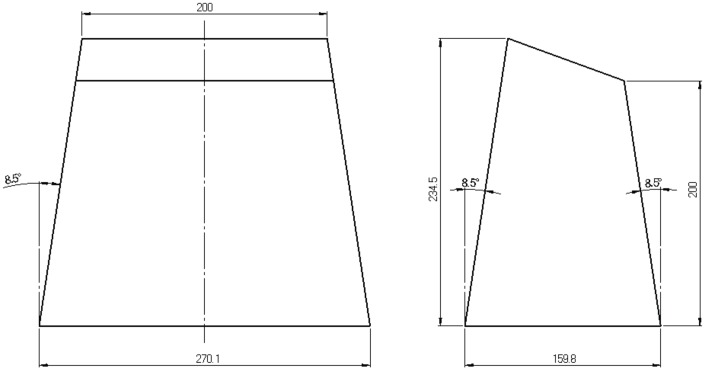
Geometry of the impact attenuator used in the case study. The truncated pyramid with rectangular base is made with plates (2.5 mm thick) welded along the edges. It is inscribed within an envelope measuring 234.5 mm × 159.8 mm × 270.1 mm.

According to the rules of the Formula SAE, one IA must be located at the forward position of the front bulkhead of the car. The IA must be at least 200 mm in both length and width, and 100 mm in height. The racing team of the University of Vigo (Spain) used for the Formula SAE 2016 the basic geometry allowed by the rules. It is the geometry of a truncated pyramid with rectangular base, and inscribed within an envelope of 234.5 mm × 159.8 mm × 270.3 mm. The forward plane was tilted (12${}^{\circ }$) in respect to the rear plane to fit to the aerodynamical front shape of the car, see Figure 1.

One IA prototype was made after two plied aluminium 5380-O plates of 2.5 mm thickness, welded together along the edges, and making a hollow structure. The total weight was 1.2 kg.

### 2.2. Impact Attenuator Test

According to the Formula SAE rules, the functional requisites of an IA for a 300 kg car, impacting with a non-yielding barrier at 7 m/s, are such that it must show an average acceleration below 20 g (we kept the typical unit system for SAE conventions, 1 g = 9.80665 m/s${}^{2}$), with a peak value below 40 g, and an absorbed energy of at least 7350 J. For the lab tests the data must be analysed according to SAE-J211 norm, which for our case included a Channel Filter Class CFC-60 (100 Hz), which smooths the data response and normalizes the results for comparison and discussion.

An energy absorption test was carried out to validate the IA using a rail-guided drop tower at a test facility (CTAG-IDIADA Safety Technology SL, Porriño, Spain). In this test, a large plate of appropriate weight (impactor) is dropped onto the part under test, fixed to a horizontal bench. For this test the impactor weight was 348 kg, and the velocity 6.47 m/s, corresponding to a drop height of 2.13 m, so its energy was equivalent to the Formula SAE specifications. For the measurements, a fixed high-speed camera (1000 FPS) and acceleration gauges (Kyowa Electronic Instruments, Tokyo, Japan) with a range of ±500 g mounted in the impactor, were used. Additionally, a thin metallic foil was used as an electrical switch to set the contact time of the impactor and as a zero reference time mark.

**Figure 2:**
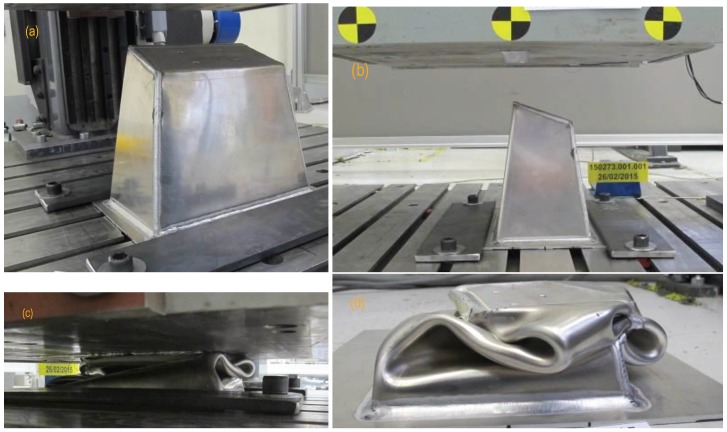
Pictures corresponding to the actual impact test. (**a**,**b**) show the Impact Attenuator fixed to the bench. The welding at the edges is clearly visible, as is the welding around the perimeter to bond the base plate. The impactor, with reference tags, is also visible; (**c**,**d**) show the piece after the test. The final height was about 60 mm.

For the test, the IA prototype was located with its longitudinal axis in vertical position, fixed to a base plate (1 mm thick) made as a frame and welded along the IA base perimeter. The plate was fixed to the bench with additional thick plates at the sides. Figure 2a,b show the test mounting and the features mentioned.

Observation of the images taken during the drop test reveals that 17 ms after the contact there was a failure of the welding at one vertical edge of the IA. This failure is expected to produce decreased rigidity in the prototype compared to the original IA design. At the end of the test, the IA was crushed to a height about 60 mm (see Figure 2c,d).

**Figure 3:**
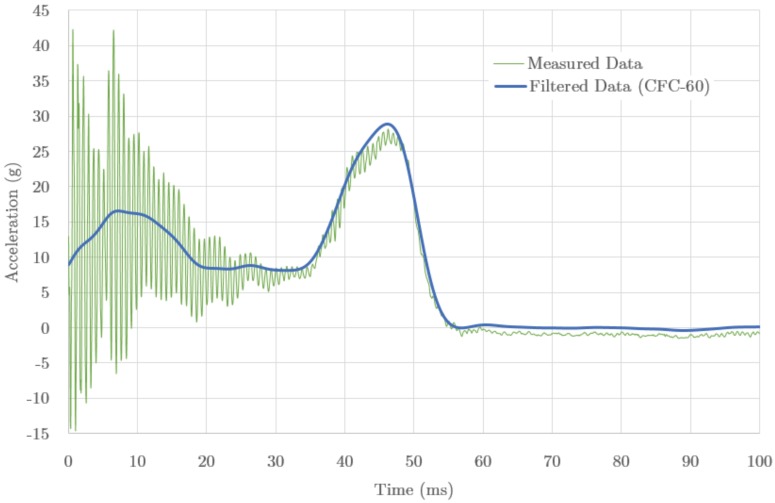
Acceleration (g) measured in the impact attenuator (IA) test, as function of time (ms), starting just after the contact mark. The measured data shows a strong oscillating pattern, which is smoothed after filtering by a CFC-60 procedure. The maximum acceleration peak reference value is 27.46 g.

The test report included information about the velocity, acceleration, displacement and energy absorption during the elapsed time of the axial crushing. Figure 3 shows the acceleration measured both as raw and filtered data. The filtered data show an average acceleration of 9.96 g, and a peak value of 28.8 g at 46 ms, corresponding to 49% and 72%, respectively, of the maximum allowed values.

The (filtered) data shows a double bumped structure, with one broad peak at about 8 ms and the main one at 46 ms, corresponding to a sequential stiffening response of the IA structure during the impact. The measured profile is caused by the initial buckling, followed by the smooth plastic yielding of the material, and a second stiffening range due to the parts self-contact caused by being crushed to the limit. The weakened stiffness caused by the weld failure contributed to make the range of flat values between the stiffening peaks more remarkable, as, otherwise, a smooth transition range would be expected.

## 3. FEM Simulation and Validation

Our first goal was to make a finite element (FE) model to reproduce the test and to validate the appropriate calculation options for the highly non-linear situation of the impact. The CAD 3D design of the IA was introduced as the geometry for ANSYS Mechanical (ANSYS Inc., Canonsburg, PA, USA), including the initial and boundary conditions, the constitutive equations and so on. The model element definition for meshing, the solver and the post-processing was done by using Ls-Dyna (Livermore Software Technology Corporation, Livermore, CA, USA) within the same framework, this being the most suitable choice for analysing our short duration event using explicit time integration.

### 3.1. Geometry and Material

The 3D geometry of the model corresponds to that used for the test, see Figure 1 and Figure 2. The impact plate was 500 mm × 500 mm, modelled with solid elements. The other parts were modelled with shell elements, with thicknesses 2.5 mm for the IA and 1 mm for the base plate. The IA and the impactor were initially placed just touching each other. In order to reduce the computational time, only the IA was defined as a flexible part, whereas the other parts defined as (infinitely) rigid components. The IA material was defined as a bi-linear material, with the plastic properties given by the tangent modulus, see Table 1. This choice, widely used and largely simplifying the model calculation, also provides enough information for describing a crush test as the one proposed. We also note that the plastic behaviour is modelled using the classical von Mises criterion for the fluency function.

We did not include any hardening factors due to strain-rate effects. The impact speed was rather slow for those effects to appear and therefore the yield limit modification is expected to be negligible [25].

### 3.2. Finite Element Meshes and Boundary and Initial Conditions

The parts defined as shell were meshed with 4-node linear quadrilateral elements. The parts defined as solid were meshed with 8-node hexahedral elements. The impactor had a structured mesh with 50 mm element edges (400 elements). The base plate had a mesh size of 25.4 mm (196 elements). These parts are of minor interest and were defined as rigid in the model. The IA was studied with different mesh sizes: 10 mm (2684 elements), 7 mm (4634 elements), 5 mm (7446 elements), and 3 mm (22,276 elements), to study the size effects. The mesh skewness was kept low (0.32) and the aspect ratio ranged from 1.46 to 2.12 for decreasing sizes.

We defined the boundary conditions according to the test conditions. The nodes of the plate were fully restricted (i.e., without degrees of freedom), and the nodes of the impactor had restricted displacements in *X* and *Y* axes in order to ensure the unique solution of the resulting discrete system. The only initial condition was the velocity of the impactor according to the test value.

According to the procedure used in ANSYS release 16, we considered the contact conditions separately. Then, we defined a bonded contact between the IA and plate at the welding edges. The contact between parts was assumed frictionless, including the self-contact of part sections happening after large deformations, which were detected automatically. This particular selection is expected to have minor influence in this study of a crushed part.

### 3.3. Analysis Settings and Solver Formulations

Due to the nature of the case study, the well-known large deformation theory is considered on the basis of the Piola-Kirchoff stress. This nonlinear behaviour coupled with the plasticity and the contact conditions make the resulting problem very difficult to be solved in an implicit way. Thus, we used the choice of explicit dynamics in LsDyna.

The explicit dynamics solver allows for the selection of the adequate formulations to integrate the defined mesh elements. The formulation used for the impactor solid elements was that of Constant Stress Solid Element. For shell elements, there are three formulations suitable for 3-dimensional quadrilateral elements: one with reduced integration (Belytschko-Tsay shell), one with full integration (Fully Integrated Shell Element), and one with thickness stretch (Belytschko-Tsay shell with thickness stretch). We can expect a greater stiffness behaviour for fully integrated elements, but all three models are a priori proper candidates. We analysed the model with the three formulations for the IA to evaluate which one is best adapted to our case. For the elements of the (rigid) base plate, we used simply the Belytschko-Tsay option. We used five points for integration through the thickness of the IA to better track the elastic and plastic states of the material within the thickness during the impact process. Only two points were used for the (rigid) base plate.

As the time progress is a critical factor for good convergence and the final quality of any explicit dynamic study, the minimum time step was defined by the well-known Courant-Friedrichs-Levy condition with a safety factor set to 0.9.

### 3.4. Simulation of the Buckling Shape

Any structure under a buckling process will deform following different modes or patterns. Those modes depend on, and are highly sensitive to, details related to weak symmetries of shape and load application, as well as to fabrication induced ’defects’ which produce weak points (after lamination, cutting, plying and welding processes) and could appear randomly distributed in the structure. As mentioned before, one welded edges broke during the test, which clearly shows the existence of real structural defects that influenced the results.

Since buckling effects are the result of an unstable equilibrium, the actual micro-defects determine the observed patterns. The simulation tool capable to answer to that situation is the artificial generation of a weak geometry in the structure, guided by the observed result. This is a quite common technique in explicit dynamic simulations [26].

**Figure 4:**
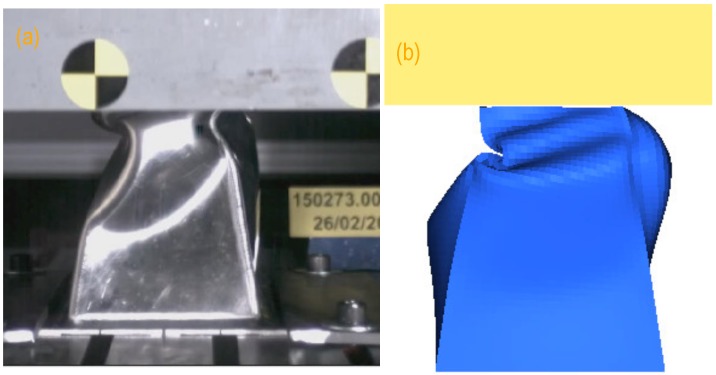

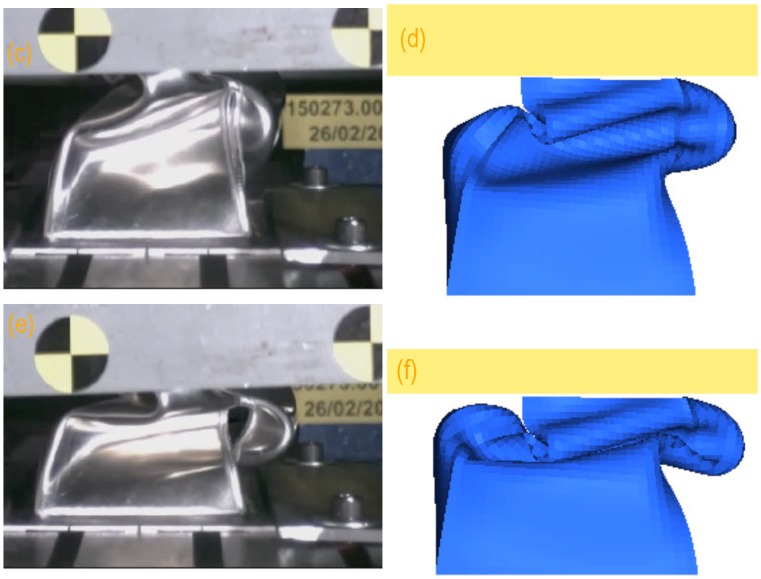
Pictures corresponding to the actual impact test (**a**,**c**,**e**) and the simulation model including a ‘failure line’ to mimic the buckling shape (**b**,**d**,**f**) for time marks (top to bottom) 28,37 and 45 ms after the impact.

In Figure 4 we show a collection of pictures taken during the IA test at different times (Figure 4a,c,e). From the beginning there is a ply inwards at the left side, which grows and defines the buckling shape. Such behaviour can only be forced in an ideal structure. We have introduced a ‘weak’ line by simply displacing the nodes of the mesh located at a certain height at that face. In that way, one ply happens in the model during the crushing (see Figure 4b,d,f). On the other hand, we were not interested in further reproducing the structure failure. That would only be representative of the test case (including a defect that caused the particular failure), and not of the general case, i.e., a better quality structure.

We have tested that the same buckling behaviour appears for any mesh size as well as for any integration procedure selected. Therefore we have a model with minimal extra input to realistically describe the observed buckling trend.

### 3.5. Mesh Size and Convergence of the Results

When solving a model in explicit dynamics, the quality of the mesh is of major importance. The (overall) size of the mesh elements makes the calculations to provide results with more details as the size decreases. Of course, the limit is imposed by the balance of the convergence of the results and the computational time involved.

In order to examine the convergence of the calculation depending on the mesh size, we performed a dedicated study. It should be noted that only the mesh of the IA part is of any interest in this study, since the other parts are just rigid, as was the case for the study of the buckling shape.

**Figure 5:**
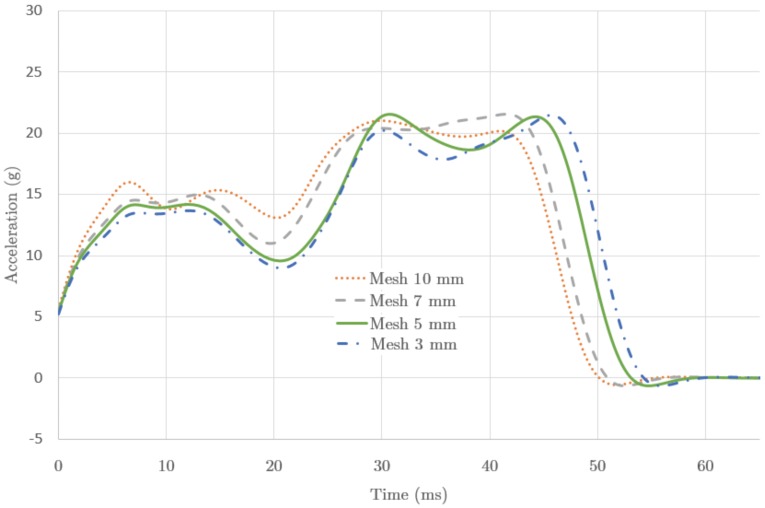
Calculated acceleration (g) as function of the time after impact with mesh sizes set to 10,7,5 and 3 mm (see legend).

Figure 5 shows plots for different results of the calculations done with the same model by just varying the mesh size of the IA part, with mesh sizes of 10,7,5 and 3 mm. It can be seen that the decreasing sizes provide converging results. The first rigidity pattern (peak) observed in the test, due to the initial plying, becomes a stable result for a size of 7 mm. The second rigidity pattern, happening at the final crushing stage, is also converging in value and peak instant with decreasing mesh sizes. For the presented results we used the full integration method. We have tested that a similar behaviour of convergence appears for any of the possible integration procedures.

The time involved in the calculation increases with the reduction of mesh size, with a factor close to 2 for reductions of 10-to-7 mm, and 7-to-5 mm, but a factor 5 for 5-to-3 mm. The limited sizes, regular geometry of the parts and mesh quality of the study allowed for small computing times (1041 s for 5 mm mesh size, for Intel Core i7™, 2.8 GHz). Considering the evolution of the results presented (see Figure 5), the mesh size with 5 mm is taken as reference for further calculations. It contains about the same quality of information as the next mesh size (3 mm), and it adds an important time economy. This becomes a limiting factor when dealing with more complex structures, and it is also important for this study considering that foam materials can demand a factor greater than 28 to perform a calculation.

### 3.6. Integration Method Dependence and Simulation Results

**Figure 6:**
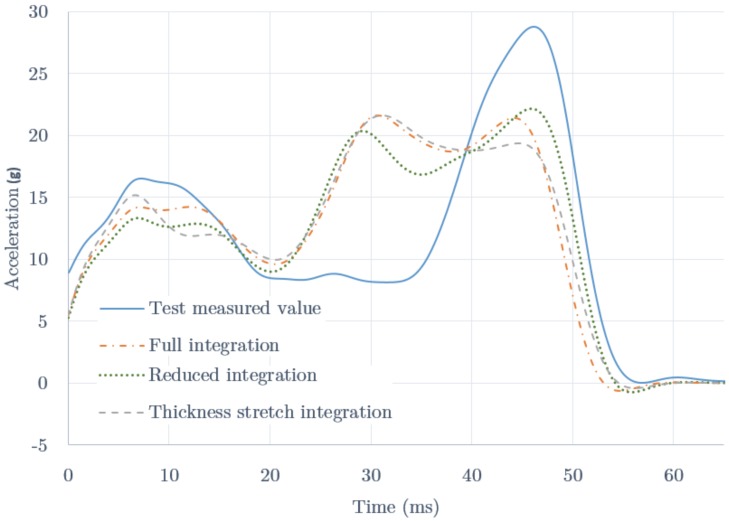
Acceleration (g) as function of the time (ms) during the crushing test. The initial time is provided by the impact switch. The data corresponds to the test measured values (solid line) and the three different formulations analyzed in our study for the integration of the mesh elements of the impact attenuator: reduced integration (dotted line), full integration (dash-dotted line) and thickness-stretch integration (dashed line).

The model described has shown itself capable of both describing the buckling shape observed and converging into a stable result according to the mesh size. We then went on to study then the influence of the different integration methods by comparing the calculations with the test results. In Figure 6 we show the time evolution of the acceleration measured in the test (solid line) and those results obtained for the different formulations of the elements of the IA as mentioned before (dotted line for reduced integration, dash-dotted line for full integration, and dashed line for thickness stretch-integration). The initial time reference is given by the contact switch.

For the first 5–8 ms, the models show an increasing stiffness just as happens in the test. We checked that this stiffening is closely related to the weak line introduced in the model (cf. Section 3.4). If not present, the initial rigidity in the models would be much higher, producing an initial decreasing acceleration pattern.

The measured data show two stiffening stages with peaks at 8 and 46 ms, and a lower strength regime in the 17–35 ms range. This behaviour is influenced by the welding failure observed during the test, which weakened the structure and produced a sharp rigidization once the plies collapsed and came into contact with each other. For the first 5–8 ms, the models also show an increasing stiffness. We can also see a peak at about 5 ms for the thickness-stretch integration method, and a broad stiffening peak around 10 ms for the other methods, which is closer to the measured data. The fact that the models do not reach the maximum value of the first peak is probably related to the initial observed offset. This is indeed related to the rigidity of the structure, and the initial buckling caused by the imperfections of the actual material.

The models produce a minimum at about 20 ms and then show a broad rigidity stage with a double peak. This pattern reproduces the collapse of the structure and the effect of the plies when they come into contact with each other. The method of thickness-stretch integration provides a narrow peak at 8 ms and high-low double peak between 30 and 50 ms, giving the less similarity to the data. The full integration method provides a broad first peak and a similar high double peak. The reduced integration method provides a low-high double peak, the second located about the same point than the test data. We selected this last method (reduced integration) as the one that better reproduces the overall stiffening pattern.

For comparison, the different methods gave peak acceleration values of 22.2 g (reduced integration), 21.6 g (full integration) and 21.6 g (thickness-stretch integration). The average accelerations were 13.8, 14.3 and 14.2 g respectively. The selected method gives the (slightly) greater acceleration peak, but also the lowest mean acceleration value.

At this point it should be noted that the measured acceleration profile, with a rather pronounced peak at the end of the crushing process, is caused by the structure failure (break) happened in the test. The important result we obtain is that a failure-free structure would provide a much smoother and more continuous acceleration profile, with peaks well below safety limits, as all simulations show.

### 3.7. Simulation of Steel Structures

It is also interesting to look into the behaviour of an IA made of high strength steel (AISI-4310), typical of automotive industry. We used different plate thicknesses and kept the external volume of the IA. The mesh models used were the same as for the aluminium, keeping the mesh size (5 mm) and the number of elements, and varying only the element thickness if necessary. The plastic properties of the steel (see Table 1) were modelled as bi-linear similarly to aluminium. The model uses reduced integration of the IA shell elements, following the previous results.

**Figure 7:**
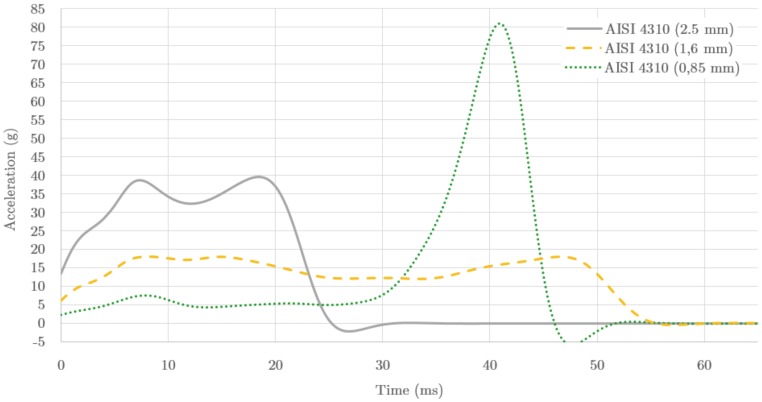
Acceleration(g) as function of the time (ms) during the impact. The data corresponds to the FE model applied to steel AISI-4310. The different results correspond to plates with thicknesses 2.5 mm (solid line), 1.6 mm (dashed line) and 0.85 mm (dotted line). The model represented is that with reduced integration of the shell IA mesh elements.

We studied different cases, whose results for the acceleration are plotted in Figure 7. The 2.5 mm thickness, like the aluminium structure with a total weight of 3.46 kg, induces large acceleration values in the initial 20 ms (solid line), reaching values close to the safety limits (40 g for Formula SAE). A thickness of 0.85 mm, a structure with a weight similar to that of aluminium (1.2 kg), has low rigidity and induces a low acceleration until crushing completely the plies and causes a large peak (dotted line), with a factor 2 out of the safety limits. A 1.6 mm thick plate (dashed line) shows a rather flat pattern below 20 g, with average of 13.8 g and a maximum peak of 17.9 g, and is within the rules. The weight of this IA is 2.3 kg, almost double of that of an aluminium one.

## 4. FEM Study of a Thermoplastic Foam

After the evaluation and comparison of our FE model, we were interested in analysing the performance of a thermoplastic material, while keeping the same geometry of the IA. For this case we used a solid bulk material, with the same shape and volume. We selected IMPAXX500™ thermoplastic foam (Dow Chemical Company, Midland, MI, USA), a high performance energy absorbing foam developed for the automotive industry.

**Figure 8:**
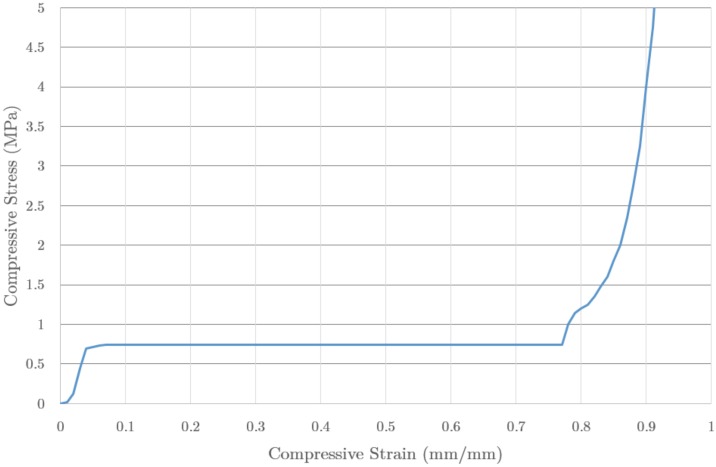
Values of stress (MPa) as function of the strain for a compressive test of IMPAXX500™ material, as given by the producer. The properties of the foam were defined as a crushable Ls-Dyna foam type, and the measured data were input into the model.

The properties of the foam were defined as for a crushable of Ls-Dyna foam type, and according to the values of stress-strain provided by the fabricant [27], plotted in Figure 8. The foam has hardly any recovery elastic behaviour and, in fact, its behaviour is highly nonlinear. The foam density is 45 kg/m${}^{3}$ and the total weight of the IA was 300 grams.

The (solid) foam was meshed with 8-node hexahedral elements of size 5 mm (76,628 elements). For the foam we used the formulation of Constant Stress Solid Element, which avoids the volumetric and shear blocking conditions in large-deformation cases, which would induce an artificial stiffening of the material behaviour. This selection needed a calculation time factor of 28 in respect to that of the aluminium (29.331 s).

Moreover, we point out that we used the same boundary and initial conditions as in the analysis performed in the case of using aluminium for the IA. Again, the large deformation theory was assumed.

## 5. Results

**Figure 9:**
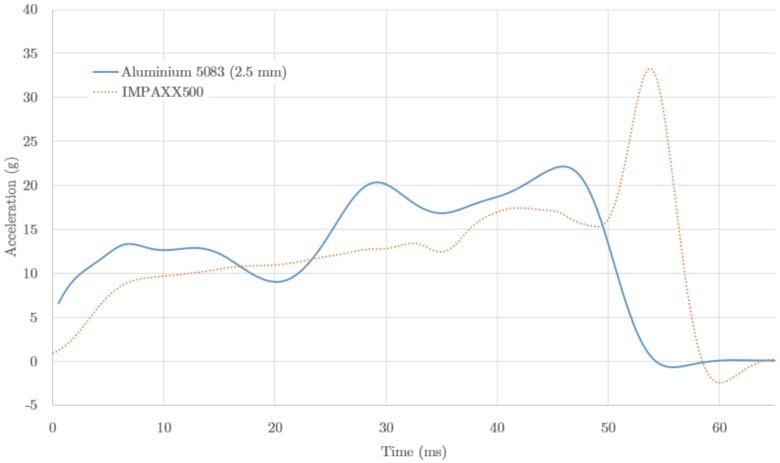
Results of the finite element (FE) analysis for the case materials. Acceleration (g) as a function of the time (ms) during the impact. The data corresponds to aluminium 5083-O (solid line) and the thermoplastic foam IMPAX500 (dotted line).

The results of the evaluated materials, aluminium and foam, were compared using the data for the acceleration time evolution, Figure 9. The foam (dotted line) shows a continuous and progressive acceleration increase corresponding to the compacting stage of the foam. Once the material has a minimum volume, a final peak appears, with a maximum well below the safety limit. It should be noted that this profile mimics the profile of the material properties (cf. Figure 8), modulated by the crushing process and the corresponding volume changes. The acceleration profiles provided peak values of 22.2 g and 33.1 g for aluminium and foam respectively, and similar average values (13.8 g and 12.3 g). In both cases the material would provide the required values for safety limits.

## 6. Conclusions

This work studied a proposed impact attenuator designed specifically for a car’s energy absorption system. It included both the test of a prototype and FE models solved with explicit dynamics. We validated one aluminium model obtained a close reproduction of the measured data when selecting the appropriate formulation of the solver. We were interested in using the realistic model to evaluate the properties of an attenuator made of different materials, including a thermoplastic foam available on the market.

The results for both aluminium and foam seem to suit the requirements of the energy absorption element perfectly. The foam show a lower initial stiffening effect, and a more regular acceleration pattern than the aluminium. Indeed the foam achieves a weight saving of 400% compared to aluminium value, and a 770% saving compared to steel.

We believe these results show that such foams are promising materials for deployment in vehicle energy absorption systems. Material and machining prices, final quality or durability were not considered for discriminating the choice of the material. It seems clear that a major overall weight saving factor could be achieved by considering the extended use of high-tech foams in other bulky parts where the safety demands are lower than those for energy absorption systems.

We also show the efficiency of the FE methodology and explicit dynamics for evaluating cases including large non-linearities. Following proper validation, the method has shown itself to be good tool for evaluating proposals for complex elements as those included in the energy absorption systems of vehicles.

## Figures and Tables

**Table 1:** Mechanical properties of the aluminium and steel used in our analysis. The values were taken after a general survey on reference providers.

Mechanical Properties	Aluminium 5083-O	Steel AISI 4310
Density (kg/m${}^{3}$)	2700	7850
Elastic Modulus (GPa)	72	200
Yielding Stregnth (MPa)	115	360
Poisson’s Coefficient	0.33	0.3
Tangent Modulus (GPa)	1.082	1.441
